# Supplementary material for: Mitofusin 2-Deficiency Suppresses Mycobacterium tuberculosis Survival in Macrophages
Source: Cells. 2019 Oct 30;8(11):1355. doi: 10.3390/cells8111355 (PMC6912353; doi:10.3390/cells8111355)
Supplement: Supplementary file 1 [file cells-08-01355-s001.pdf]

cells

MDPI

Supplementary figures

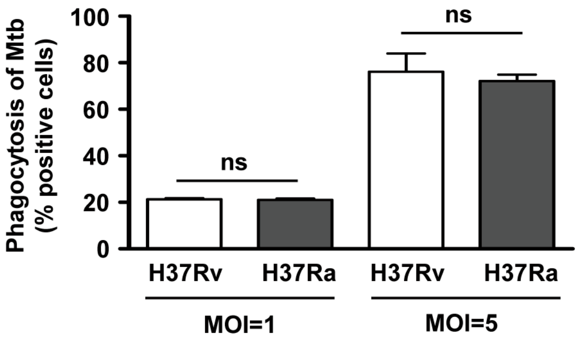

Figure S1. Phagocytosis of Mtb H37Rv or H37Ra by BMDMs.

BMDMs were infected with H37Rv or H37Ra at MOI of 1 or 5, and were incubated for 3 h. After allowing time for phagocytosis, washed with PBS to remove extracellular bacteria and then were lysed in sterile distilled water to allow intracellular bacteria to be collected. The lysates were serially diluted in plated separately onto 7H10 agar plates. The percentage of cells phagocytosing Mtb was measured by CFU analysis. All of the data are representative of three independent experiments.

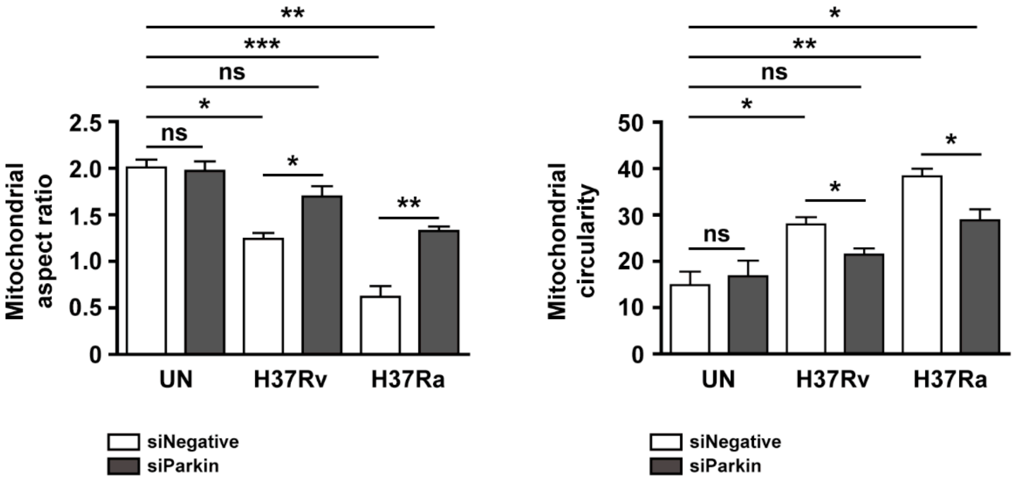

Figure S2. Quantification of mitochondrial shape parameters and aspect ratio in macrophages infected with Mtb in figure 3F.

The results are representative of three independent experiments. Statistically significant differences are indicated, as follows: *$P < 0.05$, ** $P < 0.01$, and *** $P < 0.001$.

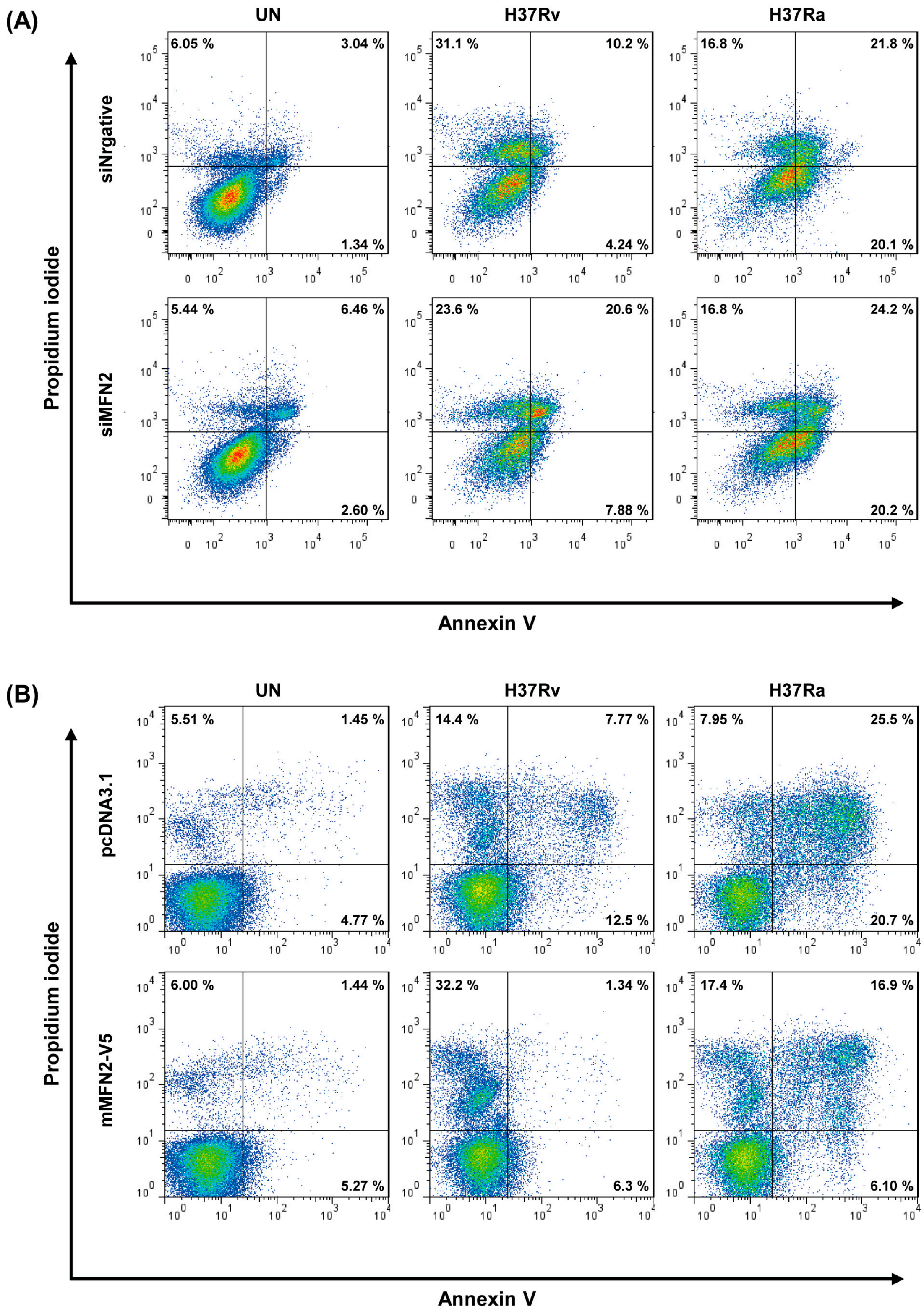

Figure S3. Flow cytometry analysis of Annexin V and propidium iodide staining in Mtb-infected macrophages.

The raw data of (A) figure 6A and (B) figure 6D. The results are representative of three independent experiments.
